# 20 Years Since the Enactment of Italian Law No. 40/2004 on Medically Assisted Procreation: How It Has Changed and How It Could Change

**DOI:** 10.3390/ijerph22020296

**Published:** 2025-02-17

**Authors:** Gianluca Montanari Vergallo, Susanna Marinelli, Gabriele Napoletano, Lina De Paola, Michele Treglia, Simona Zaami, Paola Frati

**Affiliations:** 1 Department of Anatomical, Histological, Forensic and Orthopedic Sciences, Sapienza University of Rome, 00161 Rome, Italy; gianluca.montanarivergallo@uniroma1.it (G.M.V.); gabriele.napoletano@uniroma1.it (G.N.); lina.depaola@uniroma1.it (L.D.P.); paola.frati@uniroma1.it (P.F.); 2 School of Law, Polytechnic University of Marche, 60121 Ancona, Italy; s.marinelli@pm.univpm.it; 3 Department of Biomedicine and Prevention, Section of Legal Medicine, Social Safety and Forensic Toxicology, University of Rome Tor Vergata, 00133 Rome, Italy; michele.treglia@uniroma2.it

**Keywords:** medically assisted procreation, supernumerary embryos, cryopreservation, embryo protection, same-sex parenting, surrogacy, universal crime

## Abstract

The article examines the changes to Italian legislation on assisted reproductive technology (ART) resulting from rulings by Italian courts, highlighting unresolved ethical–legal issues and potential future regulatory approaches consistent with these decisions. Additionally, it addresses the amendment defining surrogacy as “universal crime”, effective as of 18 November 2024. Through an analysis of decisions by the Constitutional Court and the Court of Cassation, it emerges that Law No. 40/2004 has been deemed unconstitutional in relation to the following: heterologous fertilization, the number of embryos that can be created, cryopreservation, the implantation of only healthy embryos, and access to ART for fertile couples. Controversial aspects include the fate of surplus embryos, access to ART for single individuals, and the recognition of parenthood for same-sex couples. The “universal crime” classification of surrogacy raises the possibility of legal consequences for individuals engaging in surrogacy abroad, even where it is lawful. Courts are unlikely to legislate on the allocation of surplus embryos without parliamentary intervention or to allow single individuals access to ART, given the perceived inconsistency with the child’s best interests. However, case-by-case evaluations are essential for recognizing non-biological or non-genetic parents in same-sex relationships and for assessing the effectiveness of the universal crime approach in safeguarding rights and public health.

## 1. Introduction

On 9 February 2004, the Italian legislature enacted Law No. 40, aiming to address reproductive issues stemming from human sterility or infertility [1,2]. While the law ostensibly sought to ensure equal rights for all parties involved in Assisted Reproductive Technology (ART) (Art. 1), it adhered closely to Catholic ethical principles [3]. This was evident in the numerous prohibitions designed to protect embryos, but such restrictions significantly limited the options for couples to become parents.

Specifically, the law prohibited singles from accessing ART, in addition to same-sex couples, fertile individuals carrying genetically transmissible diseases, and those beyond reproductive age. Article 14 imposed further restrictions, including the following: (1) the selection and destruction of embryos; (2) the cryopreservation of embryos, mandating their simultaneous transfer to the woman’s uterus during a single implantation attempt; (3) the creation of no more than three embryos per cycle of hormonal stimulation; (4) heterologous fertilization; (5) surrogacy; and (6) scientific experimentation on embryos.

The legislature intended artificial fertilization to mirror natural conception as closely as possible, requiring exactly two parents (thus excluding singles) and prohibiting more than two, as seen in surrogacy or heterologous fertilization. Furthermore, viewing the embryo as possessing a moral status akin to a human being, lawmakers sought to minimize risks of destruction, cryopreservation, or use in scientific research. The law’s strict provisions were subjected to five repeal referendums, none of which achieved the minimum voter turnout required for validity [4]. In Italy, law provisions must withstand constitutional muster, and laws can be impugned on behalf of plaintiffs by ordinary courts, which can then ask the Constitutional Court to put them to the test if there is any suspicion that such a law runs counter to constitutional precepts. If the Court strikes down law provisions based on their inconsistency with constitutional values, that is tantamount to a legislative amendment to said law. The evolution of Law 40/2004 through such a pattern has been expounded upon herein, given the fact that controversial legislations are quite difficult to amend through conventional means (i.e., by a vote of both houses of parliament). Such a way of “amending” legislation, however, is far from ideal, since it is time-consuming, exposes the plaintiffs to costly appeals, and fosters uncertainty, ultimately failing to strike a tenable balance between the rights of all the parties involved.

## 2. Materials and Methods

The authors utilized the national legal database “De Jure” and the international databases Scopus and PubMed, conducting four searches using the following keywords and search strings in titles and abstracts up to October 2024: (1) assisted reproduction AND Italian Constitutional Court; (2) assisted procreation AND Italian Constitutional Court; (3) assisted reproduction AND Italian Supreme Court (4); assisted procreation AND Italian Supreme Court. Duplicates were removed, and the full texts of relevant articles were reviewed. The investigation was extended to include pertinent works cited in the references, excluding those with exclusively medical content or otherwise unrelated to addressing the research questions outlined in the objectives.

The authors analyzed the reasoning behind court rulings to infer how unresolved issues in assisted procreation could potentially be regulated in the future.

## 3. Results

Over the years, Constitutional Court interventions have amended or repealed almost all restrictions and prohibitions. While recognizing that an embryo created with human gametes possesses a human nature, the Court deemed it unconstitutional to exclusively protect the embryo while disregarding other fundamental interests which deserve safeguards in a secular state, such as health, human dignity, equality, and the right to parenthood. Legal provisions must be framed to strike a balance between these potentially conflicting interests, without allowing one to completely override the others (Constitutional Court ruling No. 84/2016). Table 1 summarizes currently lawful ART procedures in Italy.

### 3.1. Constitutional Court Decisions

#### 3.1.1. The Number of Embryos That Can Be Created and Embryo Cryopreservation

With ruling No. 151/09, the judges, in order to strike a balance between embryo protection and the protection of the woman’s health, removed the maximum limit of three embryos to be created and then implanted (i.e., those to be transferred), as well as the requirement for a single, simultaneous implantation. This rule had forced women to undergo hormonal stimulation for every unsuccessful cycle, with all the discomfort and risks that may entail [5,6]. It thus seemed to prioritize the interests of the embryos over the woman’s. Moreover, the three-embryo limit created unequal treatment, favoring couples who could produce high-quality embryos, while discriminating against those with more significant health issues. As a result of this ruling, doctors are now required to assess, on a case-by-case basis, the number of embryos to produce based on the couple’s clinical condition, in order to offer the best chance of success, even for couples who produce lower-quality embryos. To avoid subjecting the woman to multiple cycles of hormonal stimulation, the doctor may implant only a portion of the embryos produced and cryopreserve the remaining ones [7]. It is worth noting, however, that best practices strongly recommend single-embryo transfer, whereas exceeding two is deemed unadvisable [8]. It is in fact worth highlighting the risks multiple embryo transfer poses for mothers, notably multiple pegnancies (hence a higher risk of premature birth and low birth weight) [9], and fetuses, whilst it only marginally raises the likelihood of pregnancy or live birth rates, in addition to the higher costs it entails [8,9].

#### 3.1.2. Fertile Couples and Embryo Selection

In the ruling of 14 May 2015, No. 96, the judges, in order to avoid discriminatory practices, extended the possibility of assisted reproduction to fertile couples who are carriers of genetically transmissible diseases, provided the conditions are verified by appropriate public structures and meet the criteria set for voluntary pregnancy termination (Article 6, paragraph 1, letter b, of Law No. 194/1978 and Article 4, paragraphs 3, 9, 12). Prior to this, fertile couples had to resort to so-called “procreative tourism” to achieve parenthood [10]. For fertile couples, the aim of assisted reproduction is not to address sterility or infertility, but rather to conduct diagnostic tests on in vitro embryos to ensure the birth of a healthy child. Currently, genetic testing is rather broad-ranging and can evaluate up to a thousand genetically transmissible diseases, thereby preventing the risk of rare genetic disorders, as unhealthy embryos are not implanted in the uterus [11,12]. Pre-implantation genetic diagnosis (or more commonly, pre-implantation genetic testing, PGT) is particularly relevant for women over 35/36 years old, as the rate of chromosomal abnormalities in eggs tends to increase with age, leading to a higher risk of unhealthy embryos [13]. The ability to select chromosomally healthy embryos is essential to avoid undue psychological stress for the couple. Banning PGT would jeopardize the woman’s health without protecting the embryo. Since according to Law No. 194/1978, women are entitled to abort a fetus with major genetic disorders or malformations, it must also uphold their right to know if the fetus is affected by genetic disease through PGT, as it would be illogical to implant an embryo with genetic anomalies only for it to be later aborted. It is worth remarking that PGT constitutes an essential means to prevent chromosomal aneuploidies (PGT-A) and other genetic disorders, including those that are monogenic in origin (PGT-M) or structural rearrangements (PGT-SR). While the applications of PGT are broad-ranging and may cover thousands of disorders, PGT-A is aimed at the number of chromosomes; such a form of treatment is especially valuable in case of advanced maternal age and prior recurrent miscarriage. It is therefore apparent that PGT is essential in upholding the mother’s rights under the above-mentioned Law 194/1978 and ruling 96. This ruling also reinforced the possibility of embryo cryopreservation. PGT is necessary to identify embryos that have not inherited any given genetic disease from the parent, and embryo biopsy testing inherently requires cryopreservation while awaiting results. This ruling clarified that PGT, the implantation of healthy embryos, and the cryopreservation of unhealthy embryos are all lawful. To buttress this point, a subsequent ruling by the Constitutional Court (No. 229/2015) declared unconstitutional the embryo selection ban when the doctor’s actions are “aimed at preventing the implantation of embryos affected by genetic diseases.” Having said that, any PGT or any other form of testing aimed at selecting “desirable” traits and features in the embryo, e.g., for sex selection, rather than scanning for genetic anomalies, is still illegal.

#### 3.1.3. Heterologous Fertilization

In its 10 June 2014 ruling No. 162, the Constitutional Court declared the unconstitutionality of the prohibition on the use of heterologous ART techniques (Article 4, paragraph 3, of Law No. 40/2004). The Court clarified that the inability to form a family with children alongside one’s partner, with heterologous assisted reproduction (i.e., by relying on gametes donated by a third party), can significantly impact the couple’s mental health, as happens in many other stressful situations. The Court further added that there is no risk of subjectivizing the concept of health, as mental illness can also be objectively assessed by psychiatrists [14]. Additionally, the ban on heterologous fertilization was deemed unconstitutional, because it is unreasonable and contrary to the purpose of the law stated in Article 1, i.e., to protect the rights of all parties involved. Rather than helping those with the greatest reproductive health challenges—those unable to conceive using homologous fertilization—the law prevents them from accessing heterologous methods, thus failing to meet their parenthood aspirations [15].

### 3.2. Contoversial Issues and How They Could Be Regulated

#### 3.2.1. The Disposition of Surplus Embryos

The rulings highlighted by the authors have led to fewer restrictions on assisted reproduction and embryo production [15,16]. Consequently, healthcare facilities have produced many more embryos for a larger number of people. Thus, the Court has indirectly promoted the production of supernumerary embryos, leading to a considerable increase in the number of cryopreserved embryos. In 2023, following the application of advanced assisted reproduction techniques (Level II–III), 101,035 transferable embryos were created, 39,823 (39.4%) were transferred, and 61,212 were cryopreserved, corresponding to 60.6% of the total embryos created and eligible for transfer [17]. Furthermore, out of 108,067 assisted reproduction cycles started in 2021, 34.5% (27,204) were frozen embryo transfer (FET) cycles; in the same year, 54.3% (6370) of the total live births resulting from assisted reproduction (16,625) were due to fertilization techniques using cryopreserved embryos (FET), compared to 44% (5165) of live births from fresh cycles. Lastly, with regard to Level II–III assisted reproduction techniques (92,407 cycles), the use of fresh techniques decreased proportionally, from 57.0% to 54.4% (−2.6%), while the use of FER increased from 28.4% to 29.4% (+1.0%), with oocyte thawing (FO) [18,19] techniques remaining stable at 1.6%. Expanding the scope to the European landscape, the 23rd annual report of the European IVF-Monitoring Consortium, summarizing data on assisted reproduction in 39 European countries, highlighted the notable increase in FET cycles [20]. There are various reasons for abandoning embryos. For instance, a couple may no longer need the supernumerary embryos after successfully having a child; a relationship may break down; one partner may pass away; the woman may be of advanced reproductive age [21]; family plans may change; or, following pre-implantation genetic testing, a serious hereditary condition may be detected, leading to the decision not to proceed with implantation. Despite being created for procreative purposes through IVF, supernumerary embryos are cryopreserved and stored indefinitely in specialized banks until their natural expiration [19,22,23].

Their fate depends on how the embryo is viewed [24]. Three possible solutions can be considered: the first is adoption by other individuals who offer to have the embryos implanted. IVF centers hold thousands of embryos that cannot be used, and embryo donation could encourage their use by parents who cannot afford expensive treatments. Furthermore, this would align with the protection of the embryo, which has an inherent interest in being transferred to the uterus. The second option is to donate the embryos for scientific research, with the consent of the biological parents [25]. However, Law 40 prohibits this option. This ban has been challenged on the grounds that the Italian Constitution protects the freedom of scientific research. However, the Constitutional Court has ruled that it is the responsibility of the legislature to decide whether and how cryopreserved embryos should be used: whether only those embryos affected by disease should be used—and, if so, which diseases—or even those that are scientifically “non-biopsiable”; and to determine which research objectives are significant enough to justify the sacrifice of the embryo (Constitutional Court n. 84/2016). In countries like Australia, where supernumerary embryos can be donated either to research or to other couples, undue pressure on the couple from those with an interest in either decision should be prevented [26]. Some in the field of bioethics advocate for the creation of embryos solely for scientific research purposes, but this proposal is not widely accepted, as it would risk commodifying human life, even at its prenatal stage, for the common good. The third and final option is to keep the embryos stored indefinitely, with the awareness that eventually they will no longer be viable. However, abandoning them to their fate, without any future prospect or use, is itself ethically questionable. Such a bioethical quandary arises from the complexities inherent in the determination of embryo status, legal or otherwise. While some view the embryo as a life with potential by virtue of its being an “early-stage” human life, there are broadly diverse levels of moral status which have been ascribed to human embryos. Such diverse assessments range from moral status in absolute terms, i.e., akin to fully fledged human beings, to no moral status whatsoever and therefore tantamount to other human cells. Most viewpoints however seem to be positioned in between such two extremes, as do the Italian and European legislative/regulatory frameworks governing ART [27]. In fact, most European legislation and regulations seem to agree that a progressive, gradual set of safeguards and recognition is most suitable when defining the moral status of the embryo [28,29]. Hence, an embryo’s moral value should be deemed to grow along with its biological development: such a value would therefore be relatively low at the early stages of embryogenesis, while it gradually increases as the embryo nears the fetal stage. Still, it is worth asking on what standards such a status and value ought to be based on [30]. Since even embryos at the earliest stages are acknowledged as having some degree of moral status and value, such innate traits cannot be assessed by the same standards which are commonly recognized as morally meaningful, as is the case when we acknowledge the moral status of animals, e.g., feelings, overt reactions to stimuli, the ability to feel pain, and awareness. Therefore, an early-stage embryo status is inextricably linked to its potential to develop into a fully fledged human. Although even such an approach base on “potentiality” is liable to be critiqued and called into question from a philosophical perspective [31] (and an in-depth discussion on such a debate is beyond the scope of this article), it is the most commonly cited when defining and assessing embryo status for legal and bioethical purposes [32]; in light of the above, keeping embryos in storage indefinitely could be viewed as akin to abandoning early-stage humans with no prospect of ever developing any further [33], but at the same time, an apparent paradox seems to develop, since keeping them frozen is in fact the only way to preserve that life in the first place [34]. This apparent conflict goes to show just how challenging it is to frame an effective, ethically and scientifically tenable approach for the governing of beginning-of-life issues, particularly those involving embryo status and supernumerary embryos.

#### 3.2.2. Single Individuals

Currently, the law allows access to assisted reproductive technology (ART) only for steady heterosexual couples who are stably cohabiting [34]. Thus, singles are cut off from ART access, a restriction that has increasingly been challenged.

In 2024, the Court of Florence ruled that excluding single individuals from ART violates constitutional principles, including the right to equality, health, and personal autonomy; the inviolable right to establish a family; respect for private and family life; and the right to physical and mental integrity. Furthermore, it fails to respect the freedom of self-determination regarding one’s private life, particularly the right of every individual to define their family model. The judge emphasized that, in several European countries, such as Spain, the United Kingdom, and France, assisted reproductive techniques are accessible to single women and highlighted the irrationality of a prohibition that can be circumvented through “procreative tourism”, whereby individuals seek these techniques abroad [35].

Additionally, in 2024, the Ministry of Health updated its guidelines on ART [36], aligning with judicial precedent establishing that, once fertilization has occurred, consent to ART cannot be revoked, thus enabling the woman to request the implantation of the embryo even if her partner has passed away (so-called post-mortem fertilization) [37] or if their relationship has ended [38]. In this latter scenario, the embryo is implanted after the end of stable cohabitation. In the case of post-mortem fertilization, the creation of the embryo itself occurs after the man’s death, meaning stable cohabitation no longer exists at the time of fertilization.

However, these two exceptions to the rule of stable cohabitation do not warrant eliminating the rule altogether. Allowing for embryo implantation after separation is intended to prevent embryos from remaining cryopreserved indefinitely. In the case of heterologous fertilization, there is still a clear parental intention, meaning the child will be legally recognized as the offspring of the deceased parent, even before their birth [26]. However, such cases are very rare. On the other hand, granting single individuals access to ART would mean that children would have only one legal parent, potentially leading to reduced legal protection and disadvantages compared to children with two parents. Moreover, this would represent a radical and transformative shift in the understanding of parenthood. Such a change could be welcomed by many and might result in an increase in the number of children born to single parents, although such a development is itself controversial, given the still inconclusive data regarding the upbringing of children in single-parent households as opposed to families with two parents in the house [39], as well as the many variables at play which can influence the different approaches and opinions towards single parenting.

#### 3.2.3. Same-Sex Couples

The ban on same-sex couples accessing assisted reproductive technology (ART) has not been declared unconstitutional. Article 29 of the Italian Constitution, which dates back to 1947, defines the family as a natural institution based on marriage, which at the time was understood as a union between a man and a woman. Additionally, it is within the full discretion of the legislature to grant the fundamental right to parenthood solely to heterosexual couples of childbearing age, whether married or cohabiting. According to the Court, it is the legislature’s responsibility to adapt the laws to better reflect evolving social trends, dynamics, and societal needs [34,40]. Given the current ban in Italy, same-sex couples typically travel to countries that recognize their right to become parents. After the child is born, they return with the newborn and a birth certificate that legally acknowledges both individuals as the parents, regardless of any biological connection to the child, and apply for birth registration at their local civil registry. The level of protection that judges provide to the best interests of the child depends on several factors: the composition of the couple, birthplace, the reproductive technique that was used, and the biological relationship to the child. Under Italian law, only the child of a heterosexual couple, born through either homologous or heterologous fertilization, is to be considered equivalent to a child born within marriage, and is therefore recognized by both parents (Articles 8 and 9, Law No. 40/2004).

In the case of a child conceived in Spain through assisted reproductive technology (ART), where one woman donated the egg and her partner brought the pregnancy to term, both women were considered legal parents under Spanish law. The Italian Court of Cassation ruled that “The recognition and registration of a foreign birth certificate, validly issued in Spain, in which the child is legally recognized as the child of two women, does not conflict with Italian public policy simply because Italian law does not allow or recognize such a situation. The court must respect the primary constitutional principle of the child’s best interests, which includes their right to maintain a continuous and valid legal parent–child relationship, as established abroad”.

Additionally, the court ruled that it is not permissible to oppose the recognition of the birth certificate based on a constitutional principle of public policy that prevents same-sex couples from having or raising children. Such a limitation conflicts with the fundamental right of individuals to self-determine and form a family without discrimination, just as heterosexual couples can [41].

However, if a child is conceived by two women abroad through heterologous fertilization but is born in Italy, there will be no foreign birth certificate granting the child the status of a legitimate child of the couple. Under Italian law, the newborn will have only one mother—the woman who gave birth (as per Article 269, paragraph 3, of the Civil Code). The relationship with the second parent, even if she is the egg donor, i.e., genetically related, would be protected under adoption laws in special cases (Articles 44 and following of Law No. 184/1983).

Assisted reproductive technologies (ART) have created issues regarding the recognition of parental relationships, particularly in the case of children born abroad through surrogacy, where the intended parents face challenges in securing legal recognition of their parent–child relationship [42,43,44]. In the case of a male same-sex couple, it is necessary to rely on a third woman willing to act as the surrogate mother on behalf of the couple, typically in exchange for compensation of some sort [45]. European Court of Human Rights (ECHR) case law shows a trend of progressively upholding the rights of same-sex couples, applying both Article 8 of the European Convention on Human Rights (ECHR), which protects private and family life, and Article 14 of the ECHR, which prohibits discrimination on the basis of sex, sexual orientation, or other grounds [46]. European judges have long established the primacy of social reality over biological reality, and in cases of surrogacy, they have recognized the importance of emotional bonds, even with non-biological parents [47].

The ECHR has also clarified that the “margin of appreciation” of the state includes both a surrogacy ban and the possibility of registering a foreign birth certificate for a child born through this reproductive technique. States are free to discourage “reproductive tourism” [48], but they cannot enact measures liable to deny or jeopardize the children’s rights. The ECHR further emphasizes that the state must find a way to uphold the child’s right to continue living in the family setting where they were born, without being deprived of a parental figure with whom they may have formed a consolidated relationship [49,50]. This is a complex issue that has been addressed by the United Sections of the Court of Cassation and the Constitutional Court [51]. These bodies have centered the issue on the superior interests of the child, but it is clear that it is up to the legislature to regulate this matter in detail [52,53].

The rulings identified a potential form of protection for the child through the so-called “adoption in special cases” (Articles 44 and following of Law No. 184/1983). However, this could not fully satisfy the child’s need for protection because it had limited effects; unlike “full adoption”, it did not establish parental ties with the adoptive parents’ relatives and excluded the right to inherit from them [54]. The Constitutional Court, in its ruling No. 79/2022, removed these disparities between children born through assisted reproductive technology (ART) and other children, but it once again urged the legislature to enact regulations that would provide more stable protection on this delicate and important issue, which ultimately impacts the child’s personality development and the formation of their sense of identity [55]. From the perspective of the rights of homosexual individuals, the solution of adoption in special cases is not entirely satisfactory because it forces them to go abroad to undergo ART and to turn to the court, which will assess each case individually to determine whether being adopted by a homosexual prospective parent serves the child’s best interest. This may present an element of discrimination. Though same-sex parenting is widely accepted and thoroughly acknowledged from a legal and social standpoint in most advanced democracies, in more conservative-leaning countries it may still be a rather controversial issue. Italy is in fact the only Western European country where adoptions and second-parent adoption are not open to gay couples. Legislative approaches can therefore be swayed by a host of social, moral, ethical and religious core values that make finding a broadly shared solution exceptionally hard [56]. While the different arguments in favor and against homosexual parenting are outside the main objective of this manuscript, the Italian Constitutional Court’s remarks are in our view well-balanced and sensible, in that they prioritize the child’s best interest and the essential need to maintain a family setting and project that has already been tested and consolidated [57].

## 4. Discussion

### 4.1. The New Guidelines

The Ministry of Health has released new guidelines containing recommendations for the procedures and techniques of ART, given the rate at which such technologies are developing (and the ever-broader scope of applicability) [35,58], in light of the rulings issued by the Constitutional Court. As for informed consent [59,60,61], these guidelines confirm what was laid out by Constitutional Court ruling No. 163/2024, namely that, after the assisted fertilization of the egg, consent to ART cannot be revoked, and the woman can request the implantation of the embryo even if her partner has died or if their relationship has ended [62]. In our view, the legislator, by amending Article 5, could allow single individuals, particularly single women, to access reproductive techniques, as a woman whose partner has died or who is separated is, in fact, a single woman, and, hence, the child would be raised in a single-parent family [63]. We believe there is no difference between this situation and that of a couple who separates after starting the ART procedure but still wish to fulfill their desire for parenthood. It is odd, then, to distinguish between the two cases: a child born because of assisted reproduction might suffer trauma if the biological parents break off their emotional relationship. However, for a child born following post-mortem fertilization, there is no trauma.

This possibility would allow a woman to begin a new reproductive pathway without imposing her choice on the previous partner or forming a new, stable relationship. This would not have negative consequences on the man, but it would significantly affect the psychological and physical state of the woman (who would be forced to have a child with a man who wishes to waive his right to fatherhood and with whom she no longer shares any emotional relationship), and, indirectly, it would affect the child born through ART, who would be aware that their birth was the result of a decision imposed by the mother and not a free choice of parenthood. It would therefore be preferable to allow the woman to undergo ART procedures and establish an independent parental relationship with the child, as is already the case in England, Ireland, Spain, Portugal, Germany, Greece, Belgium, Denmark, Finland, Sweden, and, more recently, France, which passed a reform to this effect in June 2021 [64]. It is worth remarking that, in the current scenario, in order to circumvent the restrictions codified in the Italian legislation, many women travel to countries where such access is allowed. This approach could be viewed as a form of violence [65]; it discriminates against women who, despite having a strong desire for motherhood, cannot afford to undertake the lengthy procedure abroad. The legislator’s decision in 2004 overlooks the principle of substantive equality, which, as is well known, requires the State to remove any social and economic obstacles that effectively hinder the full development of the human person (Article 3, second paragraph of the Constitution).

### 4.2. Surrogacy as a ‘Universal Crime’

The law, enacted by the Senate on 16 October 2024, addresses the prosecutability of the crime of surrogacy committed abroad by an Italian citizen, a relatively widespread phenomenon that has led to what is known as cross-border surrogacy [5,66,67].

Such a practice is sought by both heterosexual and homosexual couples, and is undoubtedly controversial from an ethical and legal standpoint [49,68,69], as well as considering the rights at stake, including those of the surrogate mother, which may come into conflict. These complexities are set to intensify as reproductive technologies continue to advance rapidly and are applied on an ever-larger scale, for which our current ethical, legal, and deontological criteria may prove inadequate [50,51,70,71,72,73]. The legislative innovation was introduced through an amendment to Article 12 of Law No. 40/2004, extending Italian jurisdiction to actions carried out by an Italian citizen related to the crime of surrogacy, even if committed abroad [74,75,76]. This legislative initiative has amended Article 12 of Law No. 40/2004, which, in paragraph 6, addresses crimes related to the commercialization of gametes or embryos and surrogacy. These crimes are carried out through typical actions related to their realization, organization, or publicity, and the law punishes anyone who engages in them with imprisonment ranging from three months to two years and fines ranging from 600,000 to one million euros [55,61]. A new sentence has been added to Article 12 of Law No. 40/2004, bringing the actions of an Italian citizen related to the crime of surrogacy under Italian jurisdiction, even if committed abroad. In the event of a criminal conviction, the penalties outlined in the first period will be applied [77].

### 4.3. The Criminal Liability for Acts Committed Abroad

The Italian legal system allows for the prosecution of conduct committed in a foreign country, even when that country does not classify such conduct as illegal, relying on a provision already established under Italian criminal law (Articles 7 et seq., Italian Penal Code). According to Italian criminal law, an act identified as a crime in Italy may also be punished when committed abroad, provided certain conditions are met. These conditions vary depending on whether an Italian citizen is involved as the perpetrator, an accomplice, or the victim of the crime.

Regarding the punishability of crimes committed abroad, Article 7 of the Italian Penal Code stipulates that Italian law applies both to citizens and foreigners for crimes committed abroad that are explicitly listed in the article and are characterized by significant gravity. Furthermore, it extends to any other crime for which special legal provisions or international conventions establish the applicability of Italian criminal law (Article 7, first paragraph, point 5, Penal Code) [78].

Such a provision entails the punishment under Italian criminal law of anyone who facilitates, organizes, or promotes the commercialization of gametes or surrogacy agreements, even when these actions take place abroad. This measure would theoretically put an end to cross-border surrogacy altogether, unless the intended parents choose to permanently relocate to the country where the agreement was made. The nature of the penalties would match those prescribed for crimes committed within Italian territory, regardless of whether the acts are legal in the country where they were carried out. It remains unclear, under the draft law, whether the punishment would target only Italian citizens. However, this clarification has been addressed in another related bill, Draft Bill No. 2599, which was merged with Draft Bill 306. The latest version of the draft law would limit criminal prosecution to Italian citizens [79]. If enacted, this legislative initiative would constitute a notable exception to the principle of territoriality, as defined in Article 6 of the Italian Penal Code, which stipulates that Italian law applies only when the crime is committed within Italian territory.

However, Article 7 of the Italian Penal Code codifies the possibility of applying Italian laws to crimes committed abroad, but this would only be feasible if certain conditions are met. For instance, specific legal provisions must delineate the scope of applicability of Italian law even when the act is committed outside the national territory.

In this context, the application of the law in its stricter form to cross-border surrogacy would be governed by such provisions. At present, however, it is foreseeable that future legal challenges may arise from the fact that this stricter application could conflict with some key principles of Italian criminal law. For example, the principle of dual criminality stipulates that any act punishable under Italian law may also be prosecuted when committed abroad, provided that the same act is punishable under the foreign jurisdiction’s law. Therefore, this principle would likely not apply to cross-border surrogacy, as commissioning parents typically travel to countries where surrogacy is legally practiced and explicitly regulated by national statutes.

### 4.4. The Risks to the Child’s Best Interests

Regarding the potential impact of such a proposal on the rights and well-being of children, the outlook appears rather grim. Children could potentially be separated from their parents, which would likely constitute a violation of their right to family life—particularly for those with established family structures—and would therefore conflict with Article 9 of the European Convention on Human Rights (ECHR) [48,55,61,80]. However, such extreme measures are unlikely to ever materialize. On 10 June 2023, Italy’s Minister for Family Affairs emphasized the importance of an exception to safeguard the legal recognition of family relationships for children born through surrogacy who are already part of stable and established family units. This acknowledgment highlights a growing awareness of the need to prioritize the best interests of the child over rigid adherence to prohibitive legal frameworks. However, stricter restrictions have led prosecutors to request the annulment of birth certificates previously issued for children of same-sex parents born through surrogacy abroad. On 20 June 2023, the Public Prosecutor’s Office in Padua filed an appeal to annul 33 birth certificates. Three days later, the Court of Milan ruled in favor of annulling a birth certificate that listed both the biological parent and the second intentional parent. This aligns with the jurisprudence of the Italian Court of Cassation, which has issued rulings opposing the automatic recognition of second intentional parents.

## 5. Conclusions

The Constitutional Court has significantly rewritten the rules governing fundamental aspects of assisted reproduction (ART), including heterologous fertilization, the number of embryos that can be produced, the possibility of cryopreservation, the selective implantation of healthy embryos, and access to ART for fertile couples carrying genetically transmissible diseases. Additionally, the Court of Cassation, after case-by-case assessments, has extended the possibility for same-sex couples to become parents by allowing the intentional parent to adopt a child born abroad through surrogacy. Nevertheless, the following controversial issues remain unresolved:

1. The fate of surplus embryos: Individuals suffering from incurable diseases hope for expanded research on embryos, which could lead to groundbreaking treatments.
2. Access for single individuals: Single persons argue that they are being discriminated against and denied access to ART despite the legalization of heterologous fertilization, which they seek to utilize.
3. Automatic recognition for same-sex couples: Same-sex couples aspire to an automatic recognition of parental relationships instead of undergoing the discretionary evaluation of courts during special adoption proceedings.

Given these challenges, it is unlikely that the Constitutional Court will intervene further by declaring additional provisions unconstitutional. As previously noted, the Court has emphasized that it cannot replace the judgment of Parliament with its own. It may declare a rule unconstitutional only if it is discriminatory, incompatible with the purpose of the law it is part of, or protects a single interest to the complete detriment of opposing interests. However, outside these circumstances, the Court must respect Parliament’s discretion, as it is the sole body with legislative authority.

Thus, the Constitutional Court appears to have fulfilled the role it can play in this matter. Legislative power is only vested upon the Parliament, yet lawmakers have so far shown little interest in addressing ethical questions.

The decades-long experience of trying to govern controversial and ethically sensitive social issues such as parenthood through ART cannot rely on radical ethical approaches of either side, whether rooted in Catholic doctrine or prioritizing the interests of couples at the expense of embryos/children. Such approaches are in fact likely to lead to legislative frameworks that will eventually be declared unconstitutional.

The hope, therefore, is that the legislature will acknowledge the need to revise Law No. 40/2004 and adapt it to the evolving needs of society, striving to balance all the interests involved. This would require drafting legislation that does not revolve exclusively around the concept of the “couple” but instead fosters life by safeguarding the dignity of embryos while, most importantly, ensuring that those who desire parenthood can have such a right upheld by law.

Unfortunately, not only does the surrogacy law passed on 16 October 2024 fail to protect the interests of children born through surrogacy, but it also criminalizes surrogacy even when carried out abroad. An ideal approach would involve an international effort aimed at developing a commonly shared framework, at least among countries who share a common set of values in terms of safeguards for human rights and dignity. The rights and legitimate aspirations of commissioning parents cannot conflict with the equally important rights of women in low-income countries, who are vulnerable to exploitation. The difficulties in governing such highly sensitive and complex issues are daunting, but the cost of doing nothing, or of ineffectiveness, is simply too high for us to bear. Ultimately, a truly effective set of standards must be equitable, since the right to parenthood cannot be limited to the wealthy who may travel abroad to access ART due to the unavailability of such services in their country of origin. In the absence of objective and balanced regulatory frameworks, particularly at the international level, given the inherently transnational nature of fertility traveling and cross-border surrogacy, such an approach risks jeopardizing the rights and best interests of children born through this practice, as well as the rights of parents who share a stable and genuine family plan.

## Figures and Tables

**Table 1:** Currently legal ART techniques in Italy, including their main features and restrictions.

ART Procedure	Level/Main Features/Applicable Restrictions (If Any)
Artificial fertilization	Level I; it is the most widespread technique so far.
Intrauterine insemination (IUI)	Level I
In vitro fertilization and embryo transfer	Level II; the number of embryos to be created and transferred, strictly necessary to achieve a pregnancy, is decided on a case-by-case basis for each couple, taking into account the woman’s age and her reproductive history, but it cannot exceed three. Supernumerary embryos will be cryopreserved for future attempts at implantation and pregnancy.
Intracytoplasmic sperm injection (ICSI)	Level III; a single sperm cell is injected directly into the cytoplasm of an egg, forgoing the acrosome reaction.
Gamete intrafallopian transfer (GIFT) is a tool of assisted reproductive technology against infertility.	Level III; eggs are removed from a woman’s ovaries and placed in one of the fallopian tubes, along with the man’s sperm.
Heterologous fertilization techniques	Level III; such techniques rely on third-party gamete donation. They are only legal for heterosexual couples (married or in a civil union) with documented sterility or infertility issues; singles and homosexual couples are excluded.

## Data Availability

No new data were created or analyzed in this study.
